# Depressive symptoms and dietary non-adherence among end stage renal disease patients undergoing hemodialysis therapy: systematic review

**DOI:** 10.1186/s12882-019-1622-5

**Published:** 2019-11-21

**Authors:** Mignote Hailu Gebrie, Jodi Ford

**Affiliations:** 1 University of Gondar, College of Medicine and Health Sciences, School of Nursing, Gondar, Ethiopia; 2 The Ohio State University, College of Nursing, Columbus, OH USA

**Keywords:** Depressive symptoms, Depression, Dietary adherence, Dietary non-adherence, End stage renal disease, Chronic renal disease, Nutritional status and hemodialysis

## Abstract

**Background:**

Research suggests that patients with end stage renal disease undergoing hemodialysis have a higher rate of depression and dietary non adherence leading to hospitalization and mortality. The purpose of this review was to synthesize the quantitative evidence on the relationship between depressive symptoms and dietary non adherence among end stage renal disease (ESRD) patients receiving hemodialysis.

**Methods:**

A systematic review was undertaken. Three electronic databases were searched including PubMed, CINHAL and Web of Science. Only quantitative studies published between 2001 and 2016 were included in the review.

**Result:**

A total of 141 publications were reviewed during the search process and 28 articles that fulfilled the inclusion criteria were included in the review. Eleven studies (39.3%) reported on the prevalence of depressive symptoms or depression and its effect on patient outcomes. Ten studies (35.7%) focused on dietary adherence/non adherence in patients with ESRD and the remaining seven (25%) articles were descriptive studies on the relationship between depressive symptoms and dietary non adherence in patients with ESRD receiving hemodialysis. The prevalence of depressive symptoms and dietary non adherence ranged as 6–83.49% and from 41.1–98.3% respectively. Decreased quality of life & increased morbidity and mortality were positively associated with depressive symptoms. Other factors including urea, hemoglobin, creatinine and serum albumin had also association with depressive symptoms. Regarding dietary non adherence, age, social support, educational status, behavioral control and positive attitudes are important factors in ESRD patients receiving hemodialysis. Having depressive symptoms is more likely to increase dietary non adherence.

**Conclusion:**

Depressive symptoms and dietary non adherence were highly prevalent in patients with end stage renal disease receiving hemodialysis therapy. Nearly all of the articles that examined the relationship between depressive symptoms and dietary non adherence found a significant association. Future research using experimental or longitudinal design and gold standard measures with established cut-points is needed to further explain the relationship.

## Background

End-stage renal disease (ESRD) is a growing public health problem with significant physiological, psychological and socio-economic implications for the individual, family and the community [1, 2]. Despite advances in technology and medical care, it remains a serious and life-threatening illness with a very high mortality rate and low quality of life [3–5]. Studies revealed the world wide incidence and prevalence of ESRD is increasing and it is projected that by 2020 the number of patients with ESRD will rise by nearly 60% in comparison with that of 2005 [6, 7].

There are three treatment modalities for ESRD including hemodialysis, peritoneal dialysis, and transplant. Particularly, hemodialysis, one of the most extensively used renal replacement for patients with the disease [8–11] imposes a great psychosocial burden on the patients due to the number of lifestyle, dietary, and fluid restrictions required to manage their disease [1, 2].. However, evidence indicates that patients are frequently non adherent with prescribed medications and dietary and fluid recommendations causing ongoing challenges in the care of these patients [1]. Moreover, these restrictions can impact a patient’s sense of personal control that can lead to disruptions in social relations and social withdrawal resulting in depression [2, 12–14].

In patients with ESRD, adherence to dietary restrictions is significantly associated with improved patients’ outcomes while non adherence has a risk of complication leading to death [15]. A study identified and grouped factors contributing to non-adherence as patient related, psychological, disease related, socioeconomic, therapy related and health care system related factors with their sub factors [1]. For example, in one study satisfaction with social support, comorbidity and monthly family income were factors found to be associated with dietary and fluid adherence [16]. However, others have reported discrepancies of results in studies that assessed the predictors of dietary non adherence in patients with ESRD receiving hemodialysis due primarily to the lack of a standardized method by the researchers for measuring non adherence [17].

Extant research has found that depression is the most common mental comorbidity among ESRD patients [12, 18, 19], although prevalence rates vary widely across studies ranging from 20% [20] to 90% [21]. Depressive symptoms also have been found to be associated with low adherence to prescribed dialysis treatments, including dietetic recommendations that may result in increased morbidity and mortality [2]. Despite these findings, depression remains underdiagnosed and understudied in ESRD patients [4, 22]. Depressive symptoms play a significant role in causing malnutrition that is closely related with morbidity, mortality, decrease in quality of life and delayed recovery in ESRD patients [22]. However, studies have demonstrated inconsistent findings about the relationship between depressive symptoms and fluid and dietary adherence in patients with ESRD [1]. In some studies, a negative relationship was established while in others no significant relationship was found. Therefore, the purpose of this paper is to review the prevalence of depressive symptoms and dietary non adherence in ESRD patients receiving hemodialysis and to provide a review of the quantitative research evidence regarding the relationship between depressive symptoms and dietary non adherence inpatients with ESRD receiving hemodialysis.

## Methods

### Search strategy

**Fig. 1:**
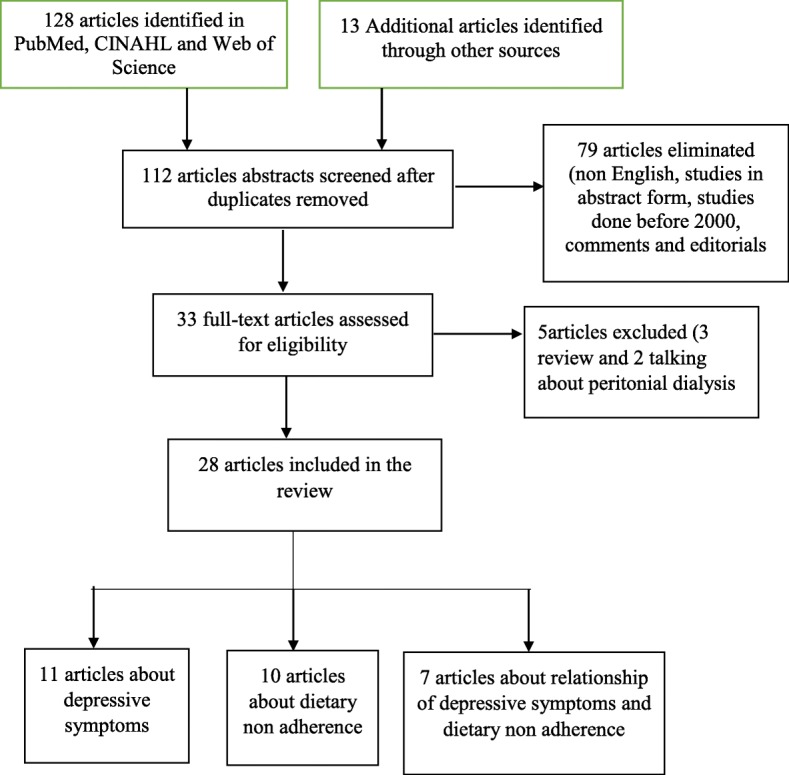
Preferred reporting items for systematic reviews and meta-analyses diagram showing the flow of information for this review

A systematic review was conducted to synthesize the different literatures regarding depressive symptoms and dietary non adherence in patients with ESRD who were receiving hemodialysis therapy. Three databases, CINAHL, PubMed and Web of Science were consulted using key words that included, “depressive symptoms”, depression, “dietary adherence/non adherence”, ESRD, “end stage renal disease”, “end stage kidney disease”, “end stage renal failure”, “nutritional status”, “dietary compliance/noncompliance” and hemodialysis. Boolean operators like “AND” and “OR” were used to combine search terms. We used MeSH terms for entry term suggestion. Bibliographies of relevant articles were also retrieved using hand searches. Search results were limited to English language articles published between 2001 and 2017, resulting in 141 articles, consisting of studies identified by electronic database search as *n* = 128 and articles identified from other searches as *n* = 13. Articles were screened for duplication and duplicates were removed *n* = 29. After removal of duplicated data, articles abstracts were screened for eligibility, which resulted in the elimination of 79 articles that were non English, studies in abstract form, studies done before 2000, comments or editorials. Finally, further screening of articles was done with application of the inclusion and exclusion criteria and critical appraisal was done by the Joanna Briggs Institute (JBI) check list (Table 1) by both authors and 28 articles that met these criteria were included in the review (Fig. 1).

**Table 1:** Joanna Briggs Institute critical appraisal checklist for analytical cross sectional studies

Sr .no	Critical appraisal checklist	Yes	No	Unclear	Not applicable
1	Were the criteria for inclusion in the sample clearly defined?	√(28)			
2	Were the study subject and the setting described in detail?	√(28)			
3	Was the exposure measure in valid and reliable way?	√(25)	√(3)		
4	Were objective, standard criteria used for measurement of the condition?	√(28)			
5	Were confounding factors identified?	√(12)		√(16)	
6	Were strategies to deal with confounding factor stated?	√(7)		√(19)	
7	Was the outcome measured in a valid and reliable way?	√(23)		√(5)	
8	Was appropriate statistical analysis used?	√(28)			

Critical appraisal

Critical appraisal for cross sectional studies was done based on Joanna Briggs Institute (JBI) check list. The appraisal showed that strategies to deal with confounding factors should be better addressed.

### Inclusion and exclusion criteria

This review included studies that were: - (1) written in English; (2) quantitative study design; (3) adults (aged 18 years or older) who were receiving hemodialysis for at least 3 months; and (4) published between 2001 and 2016. For the purpose of this review, studies that met these criteria were then categorized by types of study as 1) studies that examined the prevalence of depressive symptoms and their effect on patient outcomes, 2) studies that examined the prevalence of dietary non adherence and their effect on patient outcomes, and 3) studies that focused on the relationship between depressive symptoms and dietary non adherence. Studies were excluded if the investigators included patients with major depressive disorders.

## Results

Among 141 articles produced from the initial search 28 were included in the review. Eleven studies (39.3%) reported on the prevalence of depressive symptoms or depression and its effect on patient outcomes for those with ESRD. Ten studies (35.7%) focused on dietary adherence/non adherence in patients with ESRD and the remaining seven (25%) articles examined the relationship between depressive symptoms and dietary non adherence in patients with ESRD receiving hemodialysis.

### Depressive symptoms

Out of the eleven studies reporting on the prevalence of depressive symptoms in ESRD patients, nine (81.8%) had a sample size greater than 100 patients. The prevalence of depressive symptoms measured by self-report instruments ranged from 6 to 83.5% [18, 23–28]. Despite such discrepancies, depression was one of the most prevalent mental illnesses among hemodialysis patients [12, 18, 19].

Regarding factors associated with depressive symptoms, three (27.3%) out of eleven studies established positive associations between depressive symptoms and increased morbidity and mortality in patients with ESRD [4, 25, 29]. In three other (27.3%) studies, a significant negative correlation between depressive symptoms and quality of life was found [24, 25, 30]. In the remaining five (45.4%) studies clinical variables such as urea, hemoglobin, creatinine and serum albumin were associated with depressive symptoms. Moreover, studies found a greater likelihood of depressive symptoms among those who were younger in age, un-employed or had decreased social support [4, 23, 27]. However, inconsistent results were found for age, as old age was a significant predictor of depression in other studies [28, 30].

### Dietary non-adherence

The prevalence of dietary non adherence in ESRD patients having hemodialysis was estimated in ten (37%) of the studies included in this review. These studies used a variety of screening tools including indirect measures such as patients’ self-reports and direct measures such as pre-dialysis serum levels of potassium, phosphate, and urea nitrogen. Estimates of dietary non adherence using self-report measures ranged from 41.1–98.3% [31–35]. Non adherence ranged from 5.5 to 18% for potassium intake and from 11.7 to 25.5% for phosphorus intake [36–38].

Demographic correlates of dietary non adherence in ESRD patients having hemodialysis demonstrated a wide range of variation. Among ten studies that assessed dietary non adherence four (40%) reported younger patients were at higher-risk for non-adherence with increasing age significantly decreasing the level of non-adherence [31, 33, 34, 36]. In two (20%) studies, an association between the lack of social support (family support) and dietary non adherence was found [32, 34]. An association between smoking and dietary non adherence was reported in two (20%) studies (36, 31). Others found a positive relationship between adherence and educational status in this population [33, 37]. Education about diet and fluid restrictions and dietary counselling increase patient motivation to change and comply with dietary recommendations which in turn may improve dietary compliance [37]. Likewise, research findings demonstrated that patients with ESRD were more likely to adhere to their dietary restrictions if they had more positive attitudes and higher levels of perceived behavioral control [39].

### Depressive symptoms and dietary non adherence

Among 28 studies included in this systematic review, seven studies examined the relationship between depressive symptoms and dietary non adherence in patients with ESRD who were having hemodialysis therapy. Even though it is not clear whether depression may be the cause or the end result of malnutrition in chronic hemodialysis patients [20], studies reported a negative association between depressive symptoms/depression and dietary adherence [18, 21, 40, 41,]. A study also revealed a close association between depressive symptoms and malnutrition in this population, which may be sign of non-adherence [40]. However, a study done on Chinese hemodialysis patient relied on inter-dialytic weight gain confirmed no association between depressive symptoms and non-adherence [16].

## Discussions

### Depressive symptoms

Even though depression is the prevalent mental illness in this review, findings from each included study demonstrated discrepancy. The reason for the large variation in prevalence across studies may be due to the diverse populations’ studied and different self-report instrument used to measure depression with no consistent cut point value employed in those studies. Similarly, the difficulty in defining depression has led to challenges in its measurement.

Most (72.7%) of the studies assessed depressive symptoms using self-report instruments and only three (27.3%) used both patient self-report and physician diagnosis. Among the latter, the prevalence of physician-diagnosed depression was 13.9%, which was approximately three times lower than the prevalence for patients using self-reported Center for Epidemiologic Studies Depression Scale (CES-D) score (43.0%) [29]. Among those studies that applied patient self-report six (54.5%) used the Beck depression inventory (BDI), and the remaining five studies utilized a number of other self-report measures of depressive symptoms.

The BDI is considered to be the most commonly used and standard instrument for assessing symptoms of depression and the prevalence and severity of depression [41, 42]. However, the use of this scale in ESRD of patients is challenging due to the somatic manifestation, included in the score that may be complicated by symptoms from uremia [43]. It has been psychometrically tested and validated for ESRD patients [44]. Additionally, the measure has been found to correlate highly with the diagnostic criteria for depression, quality of life, functional status and survival over time [45]. A variety of other measures were used to assess depressive symptoms in ESRD patients receiving hemodialysis. One study employed the CES-D scale to determine the prevalence of depression in ESRD patients [24]. Even though there is a lack of studies on the validity of CES-D in this population, it has been validated against the Beck Depression Inventory and shown to have predictive power for clinical outcomes in patients with other diseases [46, 47].

Studies also used measures including the Hospital Anxiety and Depression Scale (HADS) and Geriatric Depression Scale (GDS) [23, 30]. The application of diverse instruments in these studies delivered challenges for researchers to compare findings across studies and populations, which may influence global conclusions about the prevalence of depressive symptoms in ESRD patients.

Inconsistent result was found about factors affecting depressive symptoms. This may be related to small sample sizes, patient characteristics [24, 27] and diverse countries with different cultures represented across those studies [16]. Generally, both the prevalence and factors associated with depressive symptoms showed inconsistency in these studies. In addition, the variability of screening methods for depressive symptoms made the comparison between studies challenging.

### Dietary non adherence

The prevalence of dietary non-adherence is again inconsistence. Inconsistent prevalence-rates across these studies may be the result of biological measures being affected by many factors including residual renal function, dialysis adequacy, time at which blood was obtained for the analysis between dialysis, acid-base balance, and adherence with medication [48]. Furthermore, the large variation in estimates using self-reported instruments was possibly caused by the lack of generally accepted and validated gold standard measure of adherence [39].

Six (60%) out of the ten studies employed the Dialysis Diet and Fluid Non-Adherence Questionnaire (DDFQ) [31–35, 39] while two studies utilized self-report measures other than DDFQ [36, 39]. The DDFQ was designed to measure non-adherence behavior with diet and fluid in ESRD patients having hemodialysis with established content validity in the last 14 days. Moreover, its validity could be supported by using biological measures including serum potassium, phosphorus, and BUN levels [39]. Concerning the internal consistency of DDFQ, a Cronbach’s alpha of 0.81 was reported when the measure was used with patients receiving hemodialysis [31]. Studies demonstrated that DDFQ is weakly but positively correlated with biomedical indicators of adherence, such as inter-dialytic weight gain [31, 39] whereas another study revealed no significant correlations between any of the biochemical measures of dietary adherence and self-reported dietary adherence [39].

Overall, variables such as young age, social support, educational status, behavioral control and positive attitudes were identified as factors significantly associated with dietary non adherence in ESRD patients receiving hemodialysis. In general, studies demonstrated diverse results in both the prevalence of dietary non adherence and in its related risk factors.

### Depressive symptoms and dietary non adherence

Although majority of the studies reported a negative association between depressive symptoms/depression and dietary adherence, there is still a report of no association. The inconsistent findings may be related to the use of different instruments, some studies used the Dialysis Diet and Fluid non adherence Questionnaire (DDFQ) self-reports measure [18] and others employed different biomarkers to assess dietary non adherence in these studies. For example, one study measured blood urea nitrogen (BUN), serum creatinine, potassium, and phosphate levels [49] whereas others assessed serum albumin and protein catabolic rate [20, 40, 50] as indicators of dietary non adherence. Additional issues include: different characteristics of the study samples, the use of small and convenient sampling and inclusion of a wide range of countries with different cultures. Although, these issues make study comparison more difficult, most of these researchers concluded that having depressive symptoms was associated with increased dietary non adherence. However, without considering this issues and without uniformly applying standardized criteria, establishing a causal relationship between depressive symptoms and dietary non adherence remains difficult. Moreover, since all studies included in this review are cross-sectional, the lack of temporal ordering to test if depression leads to dietary non adherence or depression results from dietary non adherence is a weakness.

## Conclusions

In conclusion, despite the variation in assessment tool applied, depressive symptoms and dietary non adherence were highly prevalent in patients with ESRD receiving hemodialysis therapy. Nearly all of the articles that examined the relationship between depressive symptoms and dietary non adherence showed significant associations. Therefore, it is possible to recommend that early diagnosis and treatment of depression and close monitoring of adherence behavior are especially important in ESRD patients to improve their quality of life. Moreover, it will be important to consider both pharmacological and non-pharmacological (e.g. social support) interventions to reduce depression in order to enhance adherence and improve survival rates. This review brought up a clear association between depressive symptoms and dietary non-adherence which enable health care providers, specifically, nurses working in nephrology unit and related areas to monitor and improve patients QOL by providing appropriate interventions. Since this review included cross sectional studies, a causal relationship between depressive symptoms and dietary non adherence among ESRD patients receiving hemodialysis cannot be determined at this time. Future research (experimental or cohort studies) using gold standard measures with established cut-points is needed to further define this relationship and explores appropriate interventions. Moreover, as this review included only quantitative studies and searched limited databases, some studies might not be included in the review.

## Data Availability

All articles retained for this review were acquired via PubMed, CINHAL and Web of science and are available to the public. All data analyzed in the present study is included in the published article.

## Abbreviations

BDI: Beck depression inventory
BUN: Blood urea nitrogen
CES-D: Center for Epidemiologic Studies Depression Scale
DDFQ: Dialysis Diet and Fluid Non-adherence Questionnaire
ESRD: End Stage Renal Disease
GDS: Geriatric Depression Scale
HADS: Hospital Anxiety and Depression Scale
